# Obstetrical outcomes and maternal morbidities associated with COVID-19 in pregnant women in France: A national retrospective cohort study

**DOI:** 10.1371/journal.pmed.1003857

**Published:** 2021-11-30

**Authors:** Sylvie Epelboin, Julie Labrosse, Jacques De Mouzon, Patricia Fauque, Marie-José Gervoise-Boyer, Rachel Levy, Nathalie Sermondade, Laetitia Hesters, Marianne Bergère, Claire Devienne, Philippe Jonveaux, Jade Ghosn, Fabienne Pessione

**Affiliations:** 1 Centre d’Assistance Médicale à la Procréation, gynécologie obstétrique, médecine de la reproduction, hôpital Bichat Claude-Bernard, AP–HP, Nord, Université de Paris, Paris, France; 2 Unilabs, direction médicale, Clichy-La-Garenne, France; 3 Université Bourgogne Franche-Comté—INSERM UMR1231, Dijon, France; 4 Service de médecine et biologie de la reproduction, hôpital Saint-Joseph, Marseille, France; 5 Inserm, équipe lipodystrophies génétiques et acquises, service de biologie de la reproduction-CECOS, Saint-Antoine research center, Sorbonne université, Paris, France; 6 Hôpital Tenon, AP–HP, Sorbonne université, Paris, France; 7 Hôpital Antoine Béclère, AP–HP, Université de Paris, Clamart, France; 8 Agence de la Biomédecine, La Plaine Saint Denis, France; 9 Service de Maladies Infectieuses et Tropicales, Hôpital Bichat Claude-Bernard, AP–HP, Nord, Université de Paris, Paris, France; 10 INSERM U1137, IAME, Faculté de Médecine site Bichat, Université de Paris, Paris, France; The University of Edinburgh Usher Institute of Population Health Sciences and Informatics, UNITED KINGDOM

## Abstract

**Background:**

To the best of our knowledge, no study has exhaustively evaluated the association between maternal morbidities and Coronavirus Disease 2019 (COVID-19) during the first wave of the pandemic in pregnant women. We investigated, in natural conceptions and assisted reproductive technique (ART) pregnancies, whether maternal morbidities were more frequent in pregnant women with COVID-19 diagnosis compared to pregnant women without COVID-19 diagnosis during the first wave of the COVID-19 pandemic.

**Methods and findings:**

We conducted a retrospective analysis of prospectively collected data in a national cohort of all hospitalizations for births ≥22 weeks of gestation in France from January to June 2020 using the French national hospitalization database (PMSI). Pregnant women with COVID-19 were identified if they had been recorded in the database using the ICD-10 (International Classification of Disease) code for presence of a hospitalization for COVID-19. A total of 244,645 births were included, of which 874 (0.36%) in the COVID-19 group. Maternal morbidities and adverse obstetrical outcomes among those with or without COVID-19 were analyzed with a multivariable logistic regression model adjusted on patient characteristics. Among pregnant women, older age (31.1 (±5.9) years old versus 30.5 (±5.4) years old, respectively, *p <* 0.001), obesity (0.7% versus 0.3%, respectively, *p <* 0.001), multiple pregnancy (0.7% versus 0.4%, respectively, *p <* 0.001), and history of hypertension (0.9% versus 0.3%, respectively, *p <* 0.001) were more frequent with COVID-19 diagnosis. Active smoking (0.2% versus 0.4%, respectively, *p <* 0.001) and primiparity (0.3% versus 0.4%, respectively, *p <* 0.03) were less frequent with COVID-19 diagnosis. Frequency of ART conception was not different between those with and without COVID-19 diagnosis (*p* = 0.28).

When compared to the non-COVID-19 group, women in the COVID-19 group had a higher frequency of admission to ICU (5.9% versus 0.1%, *p* < 0.001), mortality (0.2% versus 0.005%, *p <* 0.001), preeclampsia/eclampsia (4.8% versus 2.2%, *p <* 0.001), gestational hypertension (2.3% versus 1.3%, *p* < 0.03), postpartum hemorrhage (10.0% versus 5.7%, *p <* 0.001), preterm birth at <37 weeks of gestation (16.7% versus 7.1%, *p <* 0.001), <32 weeks of gestation (2.2% versus 0.8%, *p <* 0.001), <28 weeks of gestation (2.4% versus 0.8%, *p <* 0.001), induced preterm birth (5.4% versus 1.4%, *p <* 0.001), spontaneous preterm birth (11.3% versus 5.7%, *p <* 0.001), fetal distress (33.0% versus 26.0%, *p <* 0.001), and cesarean section (33.0% versus 20.2%, *p <* 0.001). Rates of pregnancy terminations ≥22 weeks of gestation, stillbirths, gestational diabetes, placenta praevia, and placenta abruption were not significantly different between the COVID-19 and non-COVID-19 groups. The number of venous thromboembolic events was too low to perform statistical analysis. A limitation of this study relies in the possibility that asymptomatic infected women were not systematically detected.

**Conclusions:**

We observed an increased frequency of pregnant women with maternal morbidities and diagnosis of COVID-19 compared to pregnant women without COVID-19. It appears essential to be aware of this, notably in populations at known risk of developing a more severe form of infection or obstetrical morbidities and in order for obstetrical units to better inform pregnant women and provide the best care. Although causality cannot be determined from these associations, these results may be in line with recent recommendations in favor of vaccination for pregnant women.

## Abstract 2

In a national retrospective study, Sylvie Epelboin and colleagues investigate obstetrical outcomes and maternal morbidities among pregnant women with a COVID-19 diagnosis in France.

## Author summary

**Why was this study done?:**

- In early 2020, as infection by Severe Acute Respiratory Syndrome Coronavirus 2 (SARS-CoV-2) spread worldwide, it was not known whether pregnant women with Coronavirus Disease 2019 (COVID-19) were at greater risk for maternal morbidities and adverse obstetric outcomes.
- We sought to evaluate in what extent the risk of maternal morbidities and obstetrical adverse outcomes and COVID-19 diagnosis in pregnant women could be associated.
- The possibility of having access to obstetric data of all pregnant women hospitalized in France around childbirth during the first wave of COVID-19, from January to June 2020, through access to data from the National Health Data System, allowed us to obtain results on a large representative national cohort.

**What did the researchers do and find?:**

- We conducted a retrospective analysis of a national cohort of all hospitalizations for birth ≥22 weeks of gestation occurring in France from January to June 2020, using the French national hospitalization database (PMSI).
- Maternal morbidities and adverse obstetrical outcomes with or without COVID-19 were analyzed with a model adjusted on patient characteristics that could be risk factors: maternal age, body mass index, active smoking, parity, history of diabetes or hypertension, multiple pregnancy, and assisted reproductive technique (ART) conception.
- Pregnant women with COVID-19 diagnosis were more likely to be older, have obesity, a multiple pregnancy, and history of hypertension, compared to those without COVID-19. Active smoking and primiparity were less frequent among those with COVID-19 and rates of conception using ART were similar between the 2 groups.
- When compared to the non-COVID-19 group, women in the COVID-19 group had a higher frequency of admission to intensive care unit, mortality, preeclampsia/eclampsia, gestational hypertension, peripartum and postpartum hemorrhage, preterm and very preterm spontaneous and induced birth, and cesarean section. Rates of pregnancy terminations, stillbirths, gestational diabetes, placenta praevia, placenta abruption, and venous thromboembolic events were not increased.

**What do these findings mean?:**

- These findings suggest that pregnant women with COVID-19 disease may have an increased risk of obstetrical morbidities when compared to non-COVID-19 pregnant controls.
- Results of this study may inform prevention and treatment policies for pregnant women with COVID-19.
- In clinical practice, it appears essential to be aware of these complications, notably in populations at known risk of developing a more severe form of infection or obstetrical morbidities and in order for obstetrical units to better inform pregnant women and provide the best care.
- Although causality cannot be established from our study, these findings raise the possibility that vaccination, which can be offered to pregnant women starting from the second trimester according to recommendations of several health authorities, may be useful to protect women from obstetrical excess risk, in particular in high-risk populations.

## Introduction

On December 31, 2019, a cluster of pneumonia cases of unknown cause in Wuhan, China was reported to the World Health Organization (WHO) [1]. The infection spread worldwide very quickly. Further investigations identified this infection as resulting from a new form of coronavirus, later identified by WHO as Coronavirus Disease 2019 (COVID-19), initially referred to as 2019-nCoV (2019-new coronavirus disease) and subsequently as Severe Acute Respiratory Syndrome Coronavirus 2 (SARS-CoV-2). Although most infected patients have mild pneumonia, COVID-19 can result in more severe disease, including hospitalization, admission to an intensive care unit (ICU), and death [2]. The case fatality rate of COVID-19 in Europe is estimated to be in the range of 4% to 4.5% [3]. Age and obesity (defined as body mass index (BMI) >30) have been suggested as risk factors of developing severe forms of the disease [4,5]. In France, the first confirmed case was reported on January 14, 2020. As an attempt to contain the virus, beginning on this date, all patients with confirmed COVID-19 were hospitalized, regardless of their medical condition. By March 15, 6,378 confirmed cases were reported in France, with a number of cases multiplied by 2 every 48 hours. Starting from March 15, 2020, admission in a hospital was not systematic but was based on the medical condition. Hence, patients with mild symptoms were no longer hospitalized. Hospital admissions for COVID-19 peaked on April 14, 2020, with a total of 32,131 patients hospitalized for COVID-19 in France. Approximately 83% of patients admitted to hospital for severe or critical COVID-19 were alive at Day 60 after admission [6]. In France, tests were only available in every public and private clinic starting from April 2020, then becoming systematic for all hospitalizations and births. The completeness of all COVID-19 diagnoses was not possible at the beginning of the first wave of the pandemic due to a lack of tests available.

Potential adverse effects of the virus on maternal and perinatal outcomes are of concern. SARS and MERS during pregnancy are associated with various adverse maternal and neonatal complications [7–10]. Altogether, most studies on COVID-19 have reported data from general population, but some recent studies have considered the specific situation of pregnancy. A case–control study in the United States of America comparing pregnant versus nonpregnant reproductive aged women with severe/critical COVID-19 showed that the clinical course and severity of COVID-19 in hospitalized pregnant women was worse compared to nonpregnant controls [11]. A report from the Centers of Disease Control and Prevention found that SARS-CoV-2–infected pregnant women were at higher risk of hospitalizations and adverse outcomes compared to nonpregnant women [12]. However, in this CDC report, data were not available to make the distinction between hospitalizations for COVID-19–related illness and admission for pregnancy-related conditions.

Data on pregnant women with SARS-CoV-2 suggested that the virus might increase the risk of maternal and neonatal morbidities. A surveillance study of pregnant patients hospitalized with confirmed COVID-19 infection in the United Kingdom showed that most women were in the late second or third trimester, with potential maternal adverse outcomes [13]. A systematic review and meta-analysis of 77 studies showed that, recently, pregnant women with COVID-19 diagnosed in hospital might be at increased risk of admission to an ICU, more likely to experience preterm birth and their neonates more likely to be admitted to a neonatal unit [14]. Pregnant women seem to be more frequently admitted to ICU and require more mechanical ventilation when compared to nonpregnant women [11]. Similarly, results of a meta-analysis of 2021 suggested elevated rates of ICU admission in pregnant women with COVID-19 [15]. Consistently, relatively higher rates of preterm birth, preeclampsia, and cesarean section (C-section) in case of COVID-19 infection have been reported [15,16]. Moreover, the incidence of stillbirth is significantly higher in the UK since the onset of the COVID-19 pandemic [17].

As the COVID-19 outbreak unfolds, prevention and management of COVID-19 among pregnant women and the potential maternal complications induced by the infection have become a major concern. Determining the population at risk of complications is essential in order to offer appropriate care. During our study period, in France, no clear recommendation was made for the management of pregnancies during the COVID-19 pandemic.

The aim of the present study was to determine whether maternal morbidities and adverse obstetrical outcomes were more frequent in pregnant women with COVID-19 diagnosis compared to pregnant women without COVID-19 diagnosis.

## Methods

We conducted from July to October 2020 a retrospective analysis of prospectively collected data in a national cohort of hospital births from March to June 2020. Our study did not have a prospectively developed analysis plan. The analysis of data was refined according to the successive reviews following the writing of the paper and its submission.

We compared the frequency of maternal morbidities and adverse obstetrical outcomes between pregnant women with COVID-19 diagnosis and women who gave birth during the same period with no history of hospitalization for COVID-19. The unprecedented context of the first wave of the COVID-19 pandemic made the “maternal and child health after ART” and “COVID and procreation” working groups of the Biomedicine Agency (France) initiate the study on the basis of the national register of women who gave birth during the period, without prior anticipation. This study is reported as per the Strengthening the Reporting of Observational Studies in Epidemiology (STROBE) guideline (S1 STROBE Checklist). Access to data was legally approved the National Agency of Biomedicine has an approval to access to the SNDS as notified through a decree (N°2016–1871).

### Population and data sources

All births occurring in France from January 1 to June 30, 2020, for a gestational age ≥22 weeks of gestation (whether livebirth, stillbirth, or medical terminations of pregnancy ≥22 weeks of gestation) were included in the analysis.

We used the French national hospitalization database (PMSI), included in the large French National Health System database (Système National des Données de Santé (SNDS)), in which all hospitalizations (in any public hospital or private clinic) are registered, containing information on patient characteristics, diagnoses, and treatments. Data were anonymized at data entry through a specific software making it impossible to retrieve patient identity but enabling to follow all hospitalizations through anonymized codes.

Access to PMSI data and SNDS was legally approved in accordance with French Public Health Law (decree N° 2016–1871). Access to PMSI and SNDS data for organizations that do not have permanent access or matching with other databases already available goes through an authorization procedure that involves several organizations: the National Institute for Health Data (INDS, which, in 2019, became the health data platform, the Expertise Committee for research, studies and evaluations in the field of health (CEREES)) and the Commission Nationale Informatique et Liberte (CNIL). Consequently, data are available after obtaining legal authorization (at https://www.indsante.fr/) and from the CNIL (https://www.cnil.fr/). According to the French Public Health Law, noninterventional studies on humans do not require approval from an Institutional Review Board or written consent from participants.

A declaration was made available to authorities regarding the present study, including data categories, the group of data participants, and the purpose of processing. The study was conducted according to institutional and ethical rules concerning research on tissue specimens and patients.

### COVID-19 diagnosis

Pregnant women with COVID-19 were identified if they had been recorded in the database using the ICD-10 (International Classification of Disease) code for presence of a hospitalization for COVID-19. ICD-10 codes were U07.10 for COVID-19 respiratory form, virus identified; U07.11 for COVID-19 respiratory form, unidentified virus; U07.12 for asymptomatic SARS-CoV-2 virus identified; U07.13 for other examinations and observations related to the COVID-19 pandemic (code widely used during the first weeks of the epidemic, including women with an RT-PCR positive for COVID-19, when hospitalization was systematic for any diagnosed infection, no matter how minor the symptoms, or for any diagnosis during hospitalization for obstetric reasons, while from the first half of April, only the severe forms were hospitalized, and subsequent codes aimed to refine this category); U07.14 for COVID-19 other clinical forms, virus identified; and U07.15 for COVID-19, other clinical forms, virus unidentified. The distribution of the codes relating to each case over the entire cohort “with COVID-19” is provided as a supporting information (S1 Table).

Presence of a hospitalization for COVID-19 included women having symptoms of COVID-19 associated to signs of COVID-19 on CT chest scan, as well as women with an RT-PCR and/or antigen test and/or serology test positive for COVID-19, whether symptomatic or asymptomatic. Concerning COVID-19 detection by real-time RT-PCR step and amplification after RNA extraction, different genes could be targeted depending on the commercial kit used, such as the structural protein envelope E gene specific for pan-sarbecoviruses detection or the S gene or the nonstructural ORF1a/b region specific for the SARS-CoV-2. All kits included a heterologous amplification system (Internal Control) to identify possible RT-PCR inhibition and to confirm the integrity of kit reagents.

In the COVID-19 group, more than 81% of women had a delay of less than 30 days between hospitalization with a diagnosis of COVID-19 and childbirth, of which 66% on the same day. The distribution of intervals between hospitalization for infection and hospitalization for childbirth in COVID-19 group is described in the Supporting information (S2 Table).

Due to the short period of high activity of the pandemic (February to May), a majority of women who gave birth during this period had most presumably been infected during the second or third trimester of pregnancy. The date of the onset of infection was generally unknown.

### Main indicators

Data recorded in the hospitalization database were the following: maternal age, parity, obesity (defined as BMI ≥30, and class 2 and 3 obesity as BMI ≥35), history of diabetes (type 1 or 2), history of hypertensive disorders, presence or absence of active smoking during pregnancy, mode of conception (spontaneous or assisted reproductive technique (ART)), and term of birth according to the number of weeks of gestation.

Maternal morbidity indicators were recorded via ICD codes registered during the hospitalization and included venous thromboembolism (VTE), gestational diabetes mellitus, hypertensive disorders (preeclampsia or gestational hypertension), amniotic fluid abnormalities (hydramnios, oligoamnios, infection), placenta previa, placental abruption, peripartum and postpartum hemorrhage (defined as the loss of more than 500 ml of blood within the first 22 hours following childbirth), premature rupture of membranes, and preterm birth. Preterm birth was defined as birth occurring before 37 weeks of gestation, severe preterm birth as birth occurring between 28 and 32 weeks of gestation, and very severe preterm birth as birth occurring between 22 and 27 weeks of gestation.

C-section and medical terminations of pregnancies were recorded. The only neonatal indicator recorded in the database was fetal distress. Fetal distress was identified by a single code entitled “labor and birth complicated by fetal distress, whether respiratory distress, transfer in a neonatal ICU or other.”

We also investigated the risk of hospitalization in ICU and maternal death in pregnant women with and without COVID-19.

### Statistical analysis

Characteristics of the women with and without COVID-19 diagnosis were compared using chi-squared and *t* test. Statistical relation between maternal morbidities and adverse obstetrical outcomes in the presence of COVID-19 were analyzed with univariable and multivariable logistic regression model adjusted on maternal age, BMI, active smoking, parity, history of diabetes or hypertension, multiple pregnancy, and ART conception. Statistical analysis was conducted with SAS enterprise guide V7.1. Adjusted odds ratios (aORs) and their 95% confidence intervals (CIs) were estimated.

The presence of gestational vascular disorders was assessed in women with no history of hypertension, and the presence of gestational diabetes was assessed in women with no history of diabetes.

## Results

### Maternal characteristics

A total of 244,645 births were included in the analysis, of which 874 (0.36%) were among women with COVID-19. Among women with a COVID-19 diagnosis, the time distribution of births was 130 in March, 428 in April, 224 in May, and 92 in June, respectively.

In all, 5,832 births resulted from ART, of which 16 were among women with COVID-19.

Characteristics of women with and without COVID-19 diagnosis are presented in Table 1.

**Table 1:** Maternal characteristics according to presence or absence of COVID-19 diagnosis in univariable analysis.

	All		COVID-19 diagnosis				*P*
			No		Yes		
	*n* = 244,645	%	*n =* 243,771	%	*n* = 874	%	
Mean age (y ± SD)	30.5 (±5.4)		30.5 (±5.4)		31.1 (±5.9)		<0.001
Age min–max	12–50		12–50		15–49		
≥40 y	12,088	4.9	12,023	4.9	65	7.4	<0.001
Smoking	11,807	4.8	11,785	4.8	22	2.5	<0.001
Obesity	20,722	8.5	20,579	8.4	143	16.4	<0.001
Hypertension	1,958	0.8	1,941	0.8	17	1.9	<0.001
Diabetes mellitus	1,834	0.8	1,823	0.8	11	1.3	0.08
Primiparity	104,650	42.8	104,308	42.8	342	39.1	0.03
Multiple pregnancy	4,332	1.8	4,300	1.8	32	3.7	<0.001
ART conception	5,832	2.4	5,816	2.4	16	1.8	0.28

ART, assisted reproductive technique; COVID-19, Coronavirus Disease 2019; SD, standard deviation; y, years.

Obesity: if BMI was ≥30; hypertension: if 2 values when rest exceeded 140 mm Hg.

In the COVID-19 group, women were significantly older (31.1 [std = 5.9] versus 30.5 [std = 5.4], respectively, *p* = 0.001), and a larger proportion of women were aged 40 and above (7.4% in the COVID-19 group versus 4.9% in the non-COVID-19 group, respectively). Women with COVID-19 diagnosis were more likely to have obesity, multiple pregnancy, history of hypertension (0.87% versus 0.35%, *p* = 0.001) and less likely to be actively smoking during pregnancy (0.19% versus 0.37%, *p* = 0.001) or to be primiparous (0.33% versus 0.38%, *p* = 0.03) compared to women without COVID-19 diagnosis. Frequencies of pregnancy resulting from ART were not different between those with and without COVID-19 (0.27% versus 0.36%, *p* = 0.28).

### Obstetrical complications

The rate of pregnancy terminations ≥22 weeks of gestation was 0.41% (*n* = 999/244,645) in the whole population and did not significantly differ in presence or absence of COVID-19 (*p* = 0.82). Similarly, the rate of stillbirths (*n* = 1,321/244,645, 0.54%) was not significantly different between the 2 groups in multivariable analysis (aOR = 1.60 [0.94 to 2.72], *p* = 0.08).

Maternal morbidities and adverse obstetrical outcomes of women in the COVID-19 group compared to the non-COVID-19 group are presented in Table 2.

**Table 2:** Obstetrical complications according to presence or absence of COVID-19 diagnosis^1^.

	COVID-19 diagnosis				Univariable analysis			Multivariable analysis*		
	No		Yes							
	*n* = 243,771	%	*n* = 874	%	OR	CI 95%	*P*	aOR	CI 95%	*P*
Preeclampsia	5,451	2.2	42	4.8	2.28	1.67–3.11	<0.001	2.00	1.46–2.75	<0.001
Gestational hypertension**	3,094	1.3	20	2.3	1.91	1.22–2.98	<0.001	1.65	1.05–2.58	0.03
Gestational diabetes**	29,251	12.0	139	15.9	1.40	1.17–1.68	<0.001	1.18	0.98–1.42	0.09
Hydramnios	1,662	0.68	12	1.37	2.02	1.14–3.58	0.02	1.8	1.01–3.19	0.05
Placenta praevia	1,613	0.7	<10		1.21	0.58–2.56	0.61	1.20	0.57–2.53	0.63
Placenta abruption	1,064	0.4	<10		1.31	0.54–3.17	0.54	1.26	0.52–3.04	0.61
Amniotic infection	1,360	0.6	12	1.4	2.48	1.40–4.40	0.002	2.21	1.24–3.92	0.007
Preterm birth <37 WG	17,215	7.1	146	16.7	2.64	2.21–3.16	<0.001	2.52	2.09–3.05	<0.001
32–36 WG	13,342	5.5	106	12.1	2.48	2.02–3.04	<0.001	2.45	1.98–3.04	<0.001
28–31 WG	1,947	0.8	19	2.2	3.04	1.92–4.80	<0.001	2.58	1.60–4.15	<0.001
22–27 WG	1,926	0.8	21	2.4	3.39	2.19–5.25	<0.001	2.89	1.85–4.52	<0.001
Induced preterm birth	3,367	1.4	47	5.4	4.34	3.23–5.84	<0.001	3.83	2.79–5.26	<0.001
Spontaneous preterm birth	13,848	5.7	99	11.3	2.23	1.80–2.75	<0.001	2.01	1.68–2.62	<0.001
Peripartum hemorrhage	721	0.3	8	0.9	3.11	1.55–6.27	0.002	2.94	1.46–5.92	0.003
Postpartum hemorrhage	13,874	5.7	87	10.0	1.83	1.47–2.29	<0.001	1.70	1.36–2.13	<0.001
Cesarean section	49,297	20.2	288	32.9	1.94	1.69–2.24	<0.001	1.80	1.56–2.09	<0.001
Fetal distress	63,426	26.0	288	33.0	1.40	1.21–1.61	<0.001	1.41	1.23–1.63	<0.001
ICU^2^	186	0.08	52	5.95	50.8	11.2–229.7	<0.001			
Death^2^	11	0.005	2	0.23	82.9	60.5–113.5	<0.001			

*Multivariable analysis: adjustment on maternal age, BMI, active smoking, parity, history of diabetes or hypertension, multiple pregnancy, and ART conception.

**The risk of gestational vascular disorders was assessed in women with no history of hypertension, and the risk of gestational diabetes was assessed in women with no history of diabetes.

^1^Effectives of VTE and amniotic emboly were not sufficient for a comparative analysis between populations. The incidence of amniotic embolism and VTE was too low, notably in COVID-19 population, to perform statistical analysis.

^2^These variables were not taken into account in the multivariate analysis model to avoid overfitting.

aOR, adjusted odds ratio; ART, assisted reproductive technique; BMI, body mass index; CI, confidence interval; COVID-19, Coronavirus Disease 2019; VTE, venous thromboembolism; WG, weeks of gestation.

Rates of gestational diabetes, placenta praevia, and placenta abruption were not different between the COVID-19 and non-COVID-19 groups. The incidence of amniotic embolism and VTE was too low, notably in COVID-19 population, to perform statistical analysis.

In univariable and multivariable analysis (adjusted on maternal age, BMI, active smoking, parity, history of diabetes or hypertension, multiple pregnancy, and ART conception), women in the COVID-19 group had a significantly higher frequency of preeclampsia/eclampsia (aOR = 2.0, 95% CI [1.5 to 2.8], *p* = 0.001), gestational hypertension (aOR = 1.7, 95% CI [1.1 to 2.6], *p* < 0.03), hydramnios (aOR = 1.8, 95% CI [1.0 to 3.2], *p* = 0.046), infection of amniotic fluid (aOR = 2.2, 95% CI [1.2 to 3.9], *p* = 0.007), peripartum hemorrhage (aOR = 2.9, 95% CI [1.5 to 5.9], *p =* 0.003), and postpartum hemorrhage (aOR = 1.7, 95% CI [1.4 to 2.1], *p* < 0.001), compared to the non-COVID-19 group.

In the whole population, the rate of preterm birth was 7.1% (*n* = 17,361), of which 5.5% (*n* = 13,448) were moderate preterm (32 to 36 weeks of gestation), 0.8% (*n* = 1,966) were severe preterm (28 to 31 weeks of gestation), and 0.8% (*n* = 1,947) were very severe preterm (22 to 27 weeks of gestation). In whole population, induced and spontaneous preterm birth rates were 1.4% (*n* = 3,414) and 5.7% (*n* = 13,947), respectively.

The frequency of preterm birth according to COVID-19 diagnosis is presented in Table 2. In univariable and multivariable analysis, the risk of global preterm birth was significantly increased in the COVID-19 group compared to the non-COVID-19 group (16.7% versus 7.1%, aOR = 2.52, 95% CI [2.1 to 3.1], *p* < 0.001), including severe preterm birth (aOR = 2.6, 95% CI [1.6 to 4.2]) and very severe preterm birth (aOR = 2.9, 95% CI [1.9 to 4.5], *p* < 0.001). In the COVID-19 group, the risk of medically induced preterm birth was greatly increased (aOR = 3.8, 95% CI [2.8 to 5.3], *p* < 0.001), as was the risk of spontaneous preterm birth (aOR = 2.1, 95% CI [1.7 to 2.6], *p* < 0.001).

In all, the frequency of births by C-section was 20.3% (*n* = 49,585) in the whole population. In multivariable analysis, the risk of C-section was significantly increased in the COVID-19 group, regardless of the indication (16.7% versus 7.1%, aOR = 1.8 95% CI [1.6 to 2.1], *p* < 0.001).

Fetal distress risk was also increased (aOR = 1.4, 95% CI [1.2 to 1.6], *p* < 0.001) in the COVID-19 group.

Overall, 504 women were hospitalized in ICU (0.21%), and 13 women died (0.53 per 10,000). The risk of ICU hospitalization was significantly higher in the COVID-19 group (*n =* 52/874, 5.95%) compared to the non-COVID-19 group (*n =* 186/243,771, 0.08%) (*p* < 0.001). In addition, the risk of maternal mortality was significantly higher in the COVID-19 group, with a higher rate of cases presenting pulmonary symptomatology with positive RT-PCR (*n =* 2/874, 0.23%), compared to the non-COVID-19 group (*n* = 11/243,771, 0.005%) (*p* < 0.001).

## Discussion

The experience of recent viral epidemics has led to 3 questions concerning pregnant women: (1) does COVID-19 represent a risk for pregnant women, (2) are pregnant women more affected by COVID-19 than nonpregnant women, and (3) the risk of vertical mother-to-child transmission. Our study aimed to address the question of whether COVID-19 is associated with risk of adverse obstetrical outcomes among pregnant women by comparing 2 groups of pregnant women with or without COVID-19 during the first wave of the COVID-19 pandemic.

The present study analyzed 244,645 births extracted from the PMSI occurring in France from January 1 to June 30, 2020, of which 874 women were diagnosed with COVID-19. Our results show that, in addition to a significant increase in ICU hospitalizations and maternal deaths, preeclampsia/eclampsia, gestational hypertension, hydramnios, infection of amniotic fluid, and peripartum and postpartum hemorrhage were significantly more frequent in pregnant women with COVID-19 diagnosis compared to those with no COVID-19 diagnosis. Furthermore, whether spontaneous or medically induced, the frequency of preterm birth, severe preterm birth, and very severe preterm birth was also significantly increased in the COVID-19 group. Altogether, this may explain the higher rate of C-section in the COVID-19 group compared to the non-COVID-19 group. Concerning neonatal outcomes, the frequency of fetal distress was also higher in case of COVID-19.

Before the emergence of COVID-19, coronaviruses responsible of the SARS and MERS epidemics were shown to have a negative impact on maternal and neonatal outcomes in pregnant women. Indeed, previous studies have reported higher risks of spontaneous miscarriage, preterm birth, intrauterine growth restriction, application of endotracheal intubation, admission to an ICU, renal failure, and disseminated intravascular coagulopathy in case of infection [7–10]. This new form of coronavirus, SARS-CoV-2, and its effect on pregnant women remains to be established. Our results are in line with previous literature. Notably, advanced age and having preexisting comorbidities was significantly associated with COVID-19 diagnosis, as pregnant women in the COVID-19 group of our study were significantly older and that women aged 40 and above represented a larger proportion in the COVID-19 group. In addition, we observed that obesity and history of hypertension were significantly more frequent among those with COVID-19. Consistently, increased maternal age, high BMI, chronic hypertension, and preexisting diabetes were reported to be associated with severe COVID-19 in pregnant women, and preexisting maternal comorbidity described as a risk factor for admission to an ICU and invasive ventilation [14,15]. Furthermore, a prospective analysis of 23 pregnant infected women found that 48% of infected women had preexisting comorbidities, among which morbid obesity and diabetes were the most commonly represented [16]. We also investigated whether conception by ART was more frequent among those with COVID-19 diagnosis (in the hypothesis that placental pathologies known to be increased in pregnancies obtained by ART might represent an increased risk in an infectious context) but found no differences in frequency of pregnancies resulting from ART between those with and without COVID-19. Stratifying the analysis on whether the pregnancy was spontaneous or obtained with ART was not possible due to the small number of women in the COVID-19 group whose pregnancy had been obtained by ART in our cohort (*n =* 16). In addition, our results revealed that primiparous women were underrepresented among women with COVID-19. Likewise, active smoking during pregnancy was less frequent among those in the COVID-19 group. Consistently, data from recent studies suggest that active smokers are underrepresented among women with COVID-19 in the whole population [18]. The underlying mechanisms that could explain this decreased risk remain to be established.

Moreover, our results are consistent with studies reporting potential maternal and neonatal adverse outcomes in COVID-19–positive pregnant women [13,14,16]. Allotey and colleagues’ systematic review and meta-analysis including 77 studies reported an increased risk of admission to ICU in pregnant women with SARS-CoV-2 compared to noninfected women [14]. The odds of preterm birth were high in women with COVID-19, and neonates were more likely to be admitted to a neonatal unit, which is consistent with our results showing that the risk of fetal distress was increased in case of COVID-19 [14,19]. In any case, these data have to be interpreted cautiously as the novelty of this virus and the lack of knowledge about its impact may have contributed to increased hospitalization and surveillance in case of COVID-19 diagnosis.

The risk of preterm birth is reported by all the studies with variable rates. In line with our results, the prospective study carried out by Antoun and colleagues (*n =* 23) observed relatively high rates of preterm birth, preeclampsia, and C-section in infected pregnant women [16]. In Kayem and colleagues’ multicenter study of 617 pregnancies, preterm birth was analyzed according to the estimate of the severity of COVID-19: 10.6% for moderate forms, 48.3% if oxygen was necessary, and 79.3% for severe forms [20]. Yee and colleagues’ meta-analysis [21] of 11 Chinese studies and 9,032 pregnancies showed preterm birth rate for 30% of cases, while Capobianco and colleagues’ meta-analysis [22] of 13 Chinese studies showed a slightly lower rate (23%), close to that of Han’s and colleagues’ [23] systematic review (25.3%). The meta-analysis by Diriba and colleagues [24] of 39 studies involving 1,316 pregnant women exposed 14.3% preterm birth rate. A 2021 report of the findings from the UK and USA registries of 4,005 pregnancies with SARS-CoV-2 infection were remarkably concordant. Preterm birth affected a higher proportion of women than expected based on historical and contemporaneous national data (12% in all and 16.1% of those women with confirmed infection) [25].

Nevertheless, whereas Allotey and colleagues [14] did not find an increase in other maternal complications, our results revealed in multivariable analysis that the risk of several maternal morbidities were higher in case of COVID-19, including various vascular complications such as preeclampsia/eclampsia, gestational hypertension, and peripartum and postpartum hemorrhage. The meta-analysis of Oltean and colleagues [15] found consistent results concerning preeclampsia/eclampsia.

A cohort study conducted in French Guiana by Hcini and colleagues [26], including 507 pregnancies of which 137 with COVID-19, also highlighted that the risk of postpartum hemorrhage was higher than that of patients without COVID-19.

Our results also suggest that the rate of pregnancy terminations ≥22 weeks of gestation and the rate of stillbirths did not significantly differ in presence or absence of COVID-19. In UK and USA registries, the proportions of pregnancies affected by stillbirth or early neonatal death were comparable to those in historical and contemporaneous UK and USA data, which are in line with our results [25]. Conversely, the incidence of stillbirth was reported to be significantly higher for Hcini and colleagues (OR 4.5; [1.1 to 18.6]) [26]. Similarly, Khalil and colleagues [17] found that the incidence of stillbirth was significantly higher during the pandemic period compared to during the prepandemic period (difference 6.93 [95% CI, 1.83 to 12.0] per 1,000 births; *p* = 0.01), but no direct association with COVID-19 was made. Alternatively, the authors suggest that the increased rates of stillbirths may result from indirect effects such as reluctance to go to the hospital when needed during the pandemic or that the time periods compared were not the same. Our findings for fetal distress (26%) are comparable to those described by Diriba and colleagues (26.5%) [24].

Ultimately, our study did not put in evidence higher rate of VTE complications, which may be due to the small number of events in the COVID-19 population or the possibility that patients had early anticoagulant treatment when symptomatic. However, the potential importance of VTE associated with COVID-19 must not be neglected, especially in severe forms [27,28]. Notably, the risk could be potentiated in case of pregnancy. In addition, Fabregues and colleagues recently warned about the risk of thromboembolism in women having undergone ART, as both viral infection and ovarian stimulation might be at risk of thrombotic events [29].

In all, the strengths of this study include the very high number of women who gave birth during the first wave of COVID-19 in France from February to June 2020. A large amount of data was obtained concerning complications during the third trimester of pregnancy until birth. Vaccination, which seems to be safe in pregnant women as of the second trimester according to several health authorities guidelines, is a positive perspective to protect pregnant women [30–35].

Nevertheless, this study has some limitations. The first of them was that, despite the large size of the cohort, the study was underpowered for rare outcomes as VTE and amniotic embolization. Furthermore, the presence of COVID-19 was not explored in all women in our cohort but mainly in case of symptoms (acute respiratory syndrome, fever, and flu symptoms) or obstetric complications motivating hospitalization (such as risk of preterm birth, preeclampsia, and premature rupture of membranes). Thus, asymptomatic infected women might not have been detected in our study. However, results of our study demonstrate an increase in the frequency of cases having had both maternal morbidities and COVID-19 as identified by ICD-10 codes during pregnancy. Hence, the possibility that some women with asymptomatic COVID-19 might have not been identified does not alter the observed relationship between COVID-19 diagnosis and maternal morbidities. In addition, cases of women hospitalized with COVID-19 at 38 weeks of gestation or later could not be at risk of preterm birth, and term births very early in the pandemic could not have been exposed to COVID-19 at relevant time. Similarly, these facts do not affect the observed relationship between COVID-19 diagnosis and maternal morbidities that is shown in our results. Although we had information concerning maternal hospitalizations in ICU or death, no supplementary detail was available concerning COVID-19 symptomatology. Furthermore, the date of onset of the infection was not known. On the other hand, the possibility that asymptomatic women could actually have COVID-19 does not enable to formulate any hypothesis concerning the relationship between the severity of COVID-19–related symptoms and the occurrence and severity of obstetric pathologies described. In addition, we were not able to match maternal data with neonatal data collected in another register. Finally, this study does not provide information on the possible complications during early pregnancy such as miscarriage or medical terminations of pregnancy.

## Conclusions

Overall, this large prospective national cohort study comparing the frequency of maternal morbidities of women who had a diagnosis of COVID-19 before or at the time of childbirth with that of pregnant women without diagnosis of COVID-19 who gave birth during the same period suggested an association between COVID-19 and maternal and obstetrical morbidities. We observed associations between COVID-19 diagnosis and several maternal morbidities and obstetric outcomes including preterm birth, preeclampsia, peripartum hemorrhage, and C-section. Women with COVID-19 diagnosis were more likely to be older and have obesity. The frequency of pregnancies resulting from ART was not different between the groups. In clinical practice, it appears essential to be aware of these complications, notably in populations at known risk of developing a more severe form of infection or obstetrical morbidities and in order for obstetrical units to better inform pregnant women and provide the best care. As the present study covers only women with COVID-19 diagnosed during the last second or third trimester of pregnancy, further studies are warranted to determine the impact of contracting COVID-19 during earlier stages of pregnancy.

## Supporting information

S1 STROBE Checklist STROBE statement—Checklist of items that should be included in reports of cross-sectional studies. (DOCX)

S1 Table Distribution of 874 cases of COVID-19 according to the code selected during hospitalization for infection. (DOTX)

S2 Table Intervals between hospitalization with COVID-19 diagnosis and hospitalization for childbirth in COVID-19 group (*N =* 874). (DOCX)

## Abbreviations

aOR: adjusted odds ratio
ART: assisted reproductive technique
BMI: body mass index
CI: confidence interval
CNIL: Commission Nationale Informatique et Liberte
COVID-19: Coronavirus Disease 2019
ICU: intensive care unit
SARS-CoV-2: Severe Acute Respiratory Syndrome Coronavirus 2
SNDS: Système National des Données de Santé
VTE: venous thromboembolism
WHO: World Health Organization
2019-nCoV: 2019-new coronavirus disease
