# Supplementary material for: Obstetrical outcomes and maternal morbidities associated with COVID-19 in pregnant women in France: A national retrospective cohort study
Source: PLoS Med. 2021 Nov 30;18(11):e1003857. doi: 10.1371/journal.pmed.1003857 (PMC8631654; doi:10.1371/journal.pmed.1003857)
Supplement: S1 STROBE Checklist — (DOCX) [file pmed.1003857.s001.docx]

STROBE Statement—Checklist of items that should be included in reports of ***cross-sectional studies***

|  | Item No | Recommendation | Page No |
| --- | --- | --- | --- |
| **Title and abstract** | 1 | (*a*) Indicate the study’s design with a commonly used term in the title or the abstract | Title |
|  |  | (*b*) Provide in the abstract an informative and balanced summary of what was done and what was found | Abstract  Methods and results |
| Introduction | | | |
| Background/rationale | 2 | Explain the scientific background and rationale for the investigation being reported | Introduction paragraphs 1-3 |
| Objectives | 3 | State specific objectives, including any prespecified hypotheses | Introduction, last paragraph |
| Methods | | | |
| Study design | 4 | Present key elements of study design early in the paper | Methods, paragraph “population and data sources” |
| Setting | 5 | Describe the setting, locations, and relevant dates, including periods of recruitment, exposure, follow-up, and data collection | Methods, paragraph “population and data sources” and “SARS-COV-2 infection” |
| Participants | 6 | (*a*) Give the eligibility criteria, and the sources and methods of selection of participants | Methods, paragraph “population and data sources” |
| Variables | 7 | Clearly define all outcomes, exposures, predictors, potential confounders, and effect modifiers. Give diagnostic criteria, if applicable | Methods, paragraph “main indicators” |
| Data sources/ measurement | 8* | For each variable of interest, give sources of data and details of methods of assessment (measurement). Describe comparability of assessment methods if there is more than one group | Methods, paragraph “population and data sources” and “SARS-COV-2 infection”  *Same for exposed and non-exposed groups* |
| Bias | 9 | Describe any efforts to address potential sources of bias | Methods, “”statistical analysis |
| Study size | 10 | Explain how the study size was arrived at | Methods, paragraph “population and data sources” and “SARS-COV-2 infection” |
| Quantitative variables | 11 | Explain how quantitative variables were handled in the analyses. If applicable, describe which groupings were chosen and why | Methods, “statistical analysis” |
| Statistical methods | 12 | (*a*) Describe all statistical methods, including those used to control for confounding | Methods, “statistical analysis” |
|  |  | (*b*) Describe any methods used to examine subgroups and interactions | Methods, “statistical analysis” |
|  |  | (*c*) Explain how missing data were addressed | Methods, “statistical analysis” |
|  |  | (*d*) If applicable, describe analytical methods taking account of sampling strategy | Methods, “statistical analysis” |
|  |  | (*e*) Describe any sensitivity analyses | Methods, “statistical analysis” |
| Results | | | |
| Participants | 13* | (a) Report numbers of individuals at each stage of study—eg numbers potentially eligible, examined for eligibility, confirmed eligible, included in the study, completing follow-up, and analysed | Results, “maternal characteristics”  *Same for exposed and non-exposed groups* |
|  |  | (b) Give reasons for non-participation at each stage | Results, “maternal characteristics”  *Same for exposed and non-exposed groups* |
|  |  | (c) Consider use of a flow diagram | Not applicable |
| Descriptive data | 14* | (a) Give characteristics of study participants (eg demographic, clinical, social) and information on exposures and potential confounders | Results, “maternal characteristics”  *Same for exposed and non-exposed groups* |
|  |  | (b) Indicate number of participants with missing data for each variable of interest | Results, “maternal characteristics”  *Same for exposed and non-exposed groups* |
| Outcome data | 15* | Report numbers of outcome events or summary measures | Results, “maternal characteristics” and ‘obstetrical complications”  *Same for exposed and non-exposed groups* |
| Main results | 16 | (*a*) Give unadjusted estimates and, if applicable, confounder-adjusted estimates and their precision (eg, 95% confidence interval). Make clear which confounders were adjusted for and why they were included | Results, “obstetrical complications” |
|  |  | (*b*) Report category boundaries when continuous variables were categorized | Results, “obstetrical complications” |
|  |  | (*c*) If relevant, consider translating estimates of relative risk into absolute risk for a meaningful time period | Results, “obstetrical complications” |
| Other analyses | 17 | Report other analyses done—eg analyses of subgroups and interactions, and sensitivity analyses | Results, “obstetrical complications” |
| Discussion | | | |
| Key results | 18 | Summarise key results with reference to study objectives | Discussion, paragraph 1 |
| Limitations | 19 | Discuss limitations of the study, taking into account sources of potential bias or imprecision. Discuss both direction and magnitude of any potential bias | Discussion, paragraph 6 |
| Interpretation | 20 | Give a cautious overall interpretation of results considering objectives, limitations, multiplicity of analyses, results from similar studies, and other relevant evidence | Discussion, paragraphs 1-4 |
| Generalisability | 21 | Discuss the generalisability (external validity) of the study results | In “Conclusions” |
| Other information | | | |
| Funding | 22 | Give the source of funding and the role of the funders for the present study and, if applicable, for the original study on which the present article is based | Funding disclosure |

*Give information separately for exposed and unexposed groups.

**Note:** An Explanation and Elaboration article discusses each checklist item and gives methodological background and published examples of transparent reporting. The STROBE checklist is best used in conjunction with this article (freely available on the Web sites of PLoS Medicine at http://www.plosmedicine.org/, Annals of Internal Medicine at http://www.annals.org/, and Epidemiology at http://www.epidem.com/). Information on the STROBE Initiative is available at www.strobe-statement.org.
