# Supplementary material for: Obstetrical outcomes and maternal morbidities associated with COVID-19 in pregnant women in France: A national retrospective cohort study
Source: PLoS Med. 2021 Nov 30;18(11):e1003857. doi: 10.1371/journal.pmed.1003857 (PMC8631654; doi:10.1371/journal.pmed.1003857)
Supplement: S1 Table — (DOTX) [file pmed.1003857.s002.dotx]

S1 Table: Distribution of 874 cases of COVID-19 according to the code selected during hospitalization for infection

| ICD10 codes | N |
| --- | --- |
| U07.10 : COVID-19, respiratory form, virus identified | 400 |
| U07.11 : COVID-19, respiratory form, virus unidentified | 136 |
| U07.12 : Asymptomatic SARS-CoV-2 carrier | 193 |
| U07.13 : Other examinations and observations related to the Covid-19 epidemic | 59 |
| U07.14 : COVID-19, other clinical forms, virus identified | 69 |
| U07.15 : COVID-19, other clinical forms, virus unidentified | 17 |
