# Supplementary material for: Obstetrical outcomes and maternal morbidities associated with COVID-19 in pregnant women in France: A national retrospective cohort study
Source: PLoS Med. 2021 Nov 30;18(11):e1003857. doi: 10.1371/journal.pmed.1003857 (PMC8631654; doi:10.1371/journal.pmed.1003857)
Supplement: S2 Table — (DOCX) [file pmed.1003857.s003.docx]

S2 Table: Intervals between hospitalization with COVID-19 diagnosis and hospitalization for childbirth in COVID-19 group (N=874)

Time interval N %

(Days)

| 0 | 581 | 66.5 |
| --- | --- | --- |
| 1 - 30 | 130 | 14.9 |
| 31 – 60 | 114 | 13.0 |
| 61 – 90 | 49 | 5.6 |
